# PCN-224 Nanoparticle/Polyacrylonitrile Nanofiber Membrane for Light-Driven Bacterial Inactivation

**DOI:** 10.3390/nano11123162

**Published:** 2021-11-23

**Authors:** Xiaolin Nie, Shuanglin Wu, Tanveer Hussain, Qufu Wei

**Affiliations:** 1 Key Laboratory of Eco-Textiles, Ministry of Education, Jiangnan University, Wuxi 214122, China; 18861823092@163.com (X.N.); 6170708010@stu.jiangnan.edu.cn (S.W.); 2 Textile Processing Department, Faculty of Engineering & Technology, National Textile University, Faisalabad 37610, Pakistan; hussain.tanveer@gmail.com

**Keywords:** electrospinning, antibacterial photodynamic inactivation, PCN-224, polyacrylonitrile, singlet oxygen

## Abstract

Increasing issues of pathogen drug resistance and spreading pose a serious threat to the ability to treat common infectious diseases, which encourages people to explore effective technology to meet the challenge. Photodynamic antibacterial inactivation (aPDI) is being explored for inactivating pathogens, which could be used as a novel approach to prevent this threat. Here, porphyrin-embedded MOF material (PCN-224) with photodynamic effect was synthesized, then the PCN-224 nanoparticles (NPs) were embedded into PAN nanofibers with an electrospinning process (PAN-PCN nanofiber membrane). On the one hand, polyacrylonitrile (PAN) nanofibers help to improve the stability of PCN-224 NPs, which could avoid their leakage. On the other, the PAN nanofibers are used as a support material to load bactericidal PCN-224 NPs, realizing recycling after bacterial elimination. An antibacterial photodynamic inactivation (aPDI) study demonstrated that the PAN-PCN 0.6% nanofiber membrane processed 3.00 log unit elimination towards a *E. coli* bacterial strain and 4.70 log unit towards a *S. aureus* strain under illumination. A mechanism study revealed that this efficient bacterial elimination was due to singlet oxygen (^1^O_2_). Although the materials are highly phototoxic, an MTT assay showed that the as fabricated nanofiber membranes had good biocompatibility in the dark, and the cell survival rates were all above 85%. Taken together, this work provided an application prospect of nanofibers with an aPDI effect to deal with the issues of pathogen drug resistance and spreading.

## 1. Introduction

Since we entered the 21st century, pathogens such as virus, fungi, bacteria and even parasites have become increasingly aggressive to human beings, and infectious diseases have become more violent in recent decades. More worryingly, there has been a dramatic rise in drug resistance of pathogen bacteria, posing a serious threat to our ability to deal with common pathogen infections [1,2,3]. It is reported that more than 700,000 people die of drug-resistant infection diseases each year [4], indicating that infectious diseases still remain one of the leading causes of death and illness in the world. This severe situation marks the beginning of the “post-antibiotic era”, which means a tough challenge for humans to treat infectious diseases. Faced with the aforementioned concerns, finding novel and highly efficient approaches for the treatment and prevention of infectious diseases is of great urgency.

Among many approaches to fight against pathogens, antibacterial photodynamic inactivation (aPDI) is gaining increasing attention from researchers and health care workers [5]. Unlike conventional treatments such as chemotherapy, surgery and radiotherapy, this approach causes pathogen cell damage in the presence of light, but produces little cytotoxicity in the dark. In addition to the necessary condition of light, aPDI also requires another two essential elements, photosensitizers (PSs) and oxygen (O_2_). The mechanism of aPDI inactivating pathogenic cells is due to the generation of reactive oxygen species (ROS) produced by PSs under light conditions through one or two mechanisms, including mechanism Type I and mechanism Type II. The main difference between the two mechanisms is the type of ROS produced, where mechanism Type I produces hydrogen peroxide, hydroxyl radicals or a superoxide anion radical, but the other mechanism functions by producing singlet oxygen (^1^O_2_). Pathogen inactivation by ROS-induced damage has the following important advantages [6]: i) antibiotic-resistant pathogens exhibit virtually equal levels of inactivation as their antibiotic-susceptible counterparts; ii) because of the non-specific damage caused by ROS, development of microbial resistance to aPDI is highly improbable. These advantages make aPDI particularly interesting for the prevention and transmission of pathogens, but even as these are desirable, some issues or innovations remain to be worked out. 

As one of the three key elements in aPDI, PSs play an important role in this photochemical reaction process. Traditional or common PSs, such as tetrapyrroles, phthalocyanines, phenothiazines and xanthene dyes are reported to be utilized in aPDI successfully. However, some drawbacks such as unstable photophysical properties and poor biocompatibility greatly limit their further applications as photosensitive medicines. Therefore, it is necessary to develop new PSs with superior performance. As a kind of novel and developing material, metal-organic frameworks (MOFs) have garnered much attention as a versatile nanomaterial with some advantages including easy controllability, intrinsic porosity and non-toxicity. In recent research, porphyrin-embedded MOF materials were proven to generate ROS effectively, which could kill target cancer cells [7,8]. PCN-224 (PCN is an acronym for porous coordination network), a class of porphyrin-embedded MOFs, has attracted much attention due to many desirable features for photodynamic effects, such as versatility, controllable synthesis and excellent stability to bases and acids. Most recently, PCN-224 with excellent porosity was reported to produce ^1^O_2_, and showed effective photodynamic effect in cancer therapy [9]. In our previous research, PCN-224-incorporated textiles showed effective bactericidal effects under visible light illumination, which provided novel insight into the development of the next generation light induced self-disinfecting textiles [10,11,12]. However, the pursuit of novel support fiber materials is ongoing, and using fiber materials as a support could not only load PSs during bacterial inactivation, but also recycle the PSs after use. 

Herein, as a green, facile and environmentally friendly approach, electrospinning technology was employed to fabricate PCN-224 NPs embedded polyacrylonitrile nanofiber membrane with high aPDI efficiency (PAN-PCN). This research takes advantage of the one-step method as a facile approach to synthesize PCN-224 NPs and uses the versatile electrospinning technology to fabricate nanofiber membrane, which combines nanofibers with PCN-224 NPs. On the one hand, PAN nanofiber could improve the stability of the embedded materials and prevent the leakage of the materials from the nanofibers [13]. On the other, PAN nanofibers could be used as a support material for drugs, which could be recycled after bacterial elimination. The aPDI study revealed the as fabricated PAN-PCN materials showing a broad-spectrum antibacterial behavior against Gram-negative and positive bacterial strains, such as *Escherichia coli* (*E. coli*) and *Staphylococcus aureus* (*S. aureus*). The ROS detection study indicated that light-driven ^1^O_2_ is responsible for bacterial inactivation, hence the aPDI behavior of PAN-PCN nanofiber membrane functions through a Type II mechanism. A cell viability evaluation (MTT assay) study revealed that the survival rate of L929 cells were all above 85%, meaning a reasonable biocompatible property. Taken together, this research provides a facile approach to embed PCN-224 NPs into PAN nanofibers, in order to develop a novel nanomaterial with aPDI effect to deal with the issues of pathogen spread and infectious disease. 

## 2. Experimental

### 2.1. Materials

1,3-Diphenylisobenzofuran (98%, DPBF), 5,10,15,20-tetrakis (4-carboxyphenyl) porphyrin (97%, TCPP) and zirconium oxychloride octahydrate (99%, ZrOCl_2_·8H_2_O) were obtained from Shanghai Vita Chemical Reagent Co., Ltd. (Shanghai, China) Polyacrylonitrile powder with a molecular weight of 150,000 (99.9%, PAN) was obtained from Hefei Sipin Technology Co., Ltd. (Hefei, China) N,N-dimethylformamide (99%, DMF), benzoic acid (99.5%, BA), monopotassium phosphate (99.5%, KH_2_PO_4_), sodium chloride (99.8%, NaCl), potassium chloride (99.5%, KCl), disodium hydrogen phosphate (99%, Na_2_HPO_4_), yeast extract (99.5%), plate count agar, Tween-80 (99.5%), soy peptone and tryptone were provided by Sinopharm Chemical Reagent Co., Ltd. (Shanghai, China) The above chemicals and reagents were used as received. Ultrapure water was sterilized before use.

### 2.2. Synthesis of Porous Coordination Network (PCN)-224 Nanoparticles (NPs)

PCN-224 NPs were synthesized by a one pot solvothermal method. In a typical procedure, 2.8 g BA (23 mmol), 0.1 g TCPP (0.13 mmol) and 0.3 g ZrOCl_2_·8H_2_O (0.93 mmol) were successively added into DMF (100 mL). After 10 min of sonication, the solution was heated by an oil bath at 90 °C with strong magnetic stirring. After nucleation for 5 h, the solution was placed at room temperature. When the solution temperature was cooled down, PCN-224 NPs were centrifuged for 30 min (11,525× g). Then, the separated PCN-224 NPs were washed with fresh DMF and ethanol. At last, the freeze-dried PCN-224 NPs were kept in the dark.

### 2.3. Fabrication of Polyacrylonitrile (PAN)-PCN Nanofiber Membrane

PAN powder and PCN-224 NPs were successively added into DMF, resulting in a solution of 11% wt%, then the polymer solution was stirred overnight at room temperature. The well-dispersed solution was subsequently used for electrospinning. The weight ratios of PCN-224 NPs in PAN polymer were 0 wt%, 0.1 wt%, 0.3 wt% and 0.6 wt%.

The electrospinning parameters were set as follows: the feeding speed from the syringe was 0.7 mL/h; the applied voltage was 11.5 kV; the collecting distance between the collector and the needle was about 15 cm. After the same period of electrospinning, the fabricated composite membranes were named as PAN-PCN X%, where X represents the weight percentage of PCN-224 NPs. Finally, the membranes were sealed and kept away from light.

### 2.4. Characterization

The nanofiber surface morphologies and EDS maps of as fabricated samples were collected and observed by using a SU8010 SEM (Hitachi) equipped with IXRF SYSTEMS energy-dispersive spectroscopy. Before observation, membrane samples were gold sputtered on an aluminum stub. XRD spectrum collection at a speed of 0.2 s per step from 3° to 30° by a Bruker D2 PHASER instrument was performed to analyze the structure of the materials. FT-IR spectra scanning from 4000 to 500 cm^−1^ were collected by a Nicolet iS 10 Nicolet Nexus spectrometer. UV-visible absorbance results of PCN-224 and PAN-PCN nanofiber membranes were collected by a UV-2600 Shimadzu spectrophotometer. Thermal behavior analysis including TGA and DTG was performed by a Mettler Toledo thermogravimetric instrument. Under a nitrogen environment, the heating rate was set 10 °C per min.

### 2.5. Antibacterial Activity Evaluation

*Escherichia coli* (ATCC-8099) and *Staphylococcus aureus* (ATCC-6538) are typical Gram-negative and positive bacterial strains, which are widely distributed in the environment. The two bacterial strains were selected to evaluate the aPDI performance of as fabricated PAN-PCN X% nanofiber membranes. In a typical procedure, for *E. coli*, a bacterial strain was cultured in lysogeny broth (LB) for 8–10 h at 37 °C in a constant temperature shaker (120 rpm). For *S. aureus*, a bacterial strain was cultured in the same condition, but replacing LB with tryptic soy broth (TSB). Optical density measurement at 600 nm was used to detect the concentration of cultures (OD600), the initial concentration of about 1–5 × 10^8^ colony forming units (CFU/mL) was used for sequent operation. Cultures were centrifuged for 10 min to remove LB or TSB (10,000 rpm) and then added into fresh PBS. LB, TSB and PBS solution used in the operation were sterilized before the experiment. PBS solution included Na_2_HPO_4_ (10 mM), NaCl (170 mM), KH_2_PO_4_ (1.8 mM), 3.4 mM KCl (1.8 mM) and Tween-80 (0.05%), and pH was adjusted to 7.4.

Upon the preparation of bacterial PBS solution, a 500 W Xenon arc lamp (λ ≥ 420 nm) equipped with a long-pass optical filter was provided for aPDI study, and the vertical distance was set to 12 cm.

Sterile 24-well plates were used to perform the aPDI study. In a typical procedure, PAN-PCN X% nanofiber membrane with a diameter of 14 mm was first added into a well and then 0.1 mL of PBS solution containing bacterial cells was deposited on the membrane surface. The 24-well plate containing PAN-PCN X% nanofiber membrane and bacteria was kept away from light for 60 min at room temperature to make sure that the bacteria cells are in full contact with the material (60 min of pre-incubation). After pre-incubation, the 24-well plate was subjected to illumination provided by a Xe lamp. The above studies were performed in triplicate including experimental groups and control groups. 

After illumination, sterilized absolute PBS solution (0.9 mL) was added to each well of the experimental and control groups. After shaking slightly, bacterial PBS solution (0.1 mL) was taken from each well, and diluted by a factor of 10 in an Eppendorf tube (1.5 mL). Then, the 10-fold dilution was performed six times in total. After dilution, every single series in the Eppendorf tubes was plated on the surface of culture plates (*E. coli* cells cultured using LB-agar culture plates and *S. aureus* cells using TSB-agar culture plates). The plates were immediately transferred into a constant temperature incubator away from light at 37 °C for about 15–24 h until bacterial colonies were visible to the naked eye. An unpaired Student’s two-tailed *t*-test was applied to make the statistical significance.

### 2.6. Singlet Oxygen (^1^O_2_) Detection

PCN-224 NPs were reported to generate ^1^O_2_ when exposed to light illumination, which produced photodynamic effects. Hence, the generation of ^1^O_2_ of the PAN-PCN 0.6% nanofiber membrane was identified here. A piece of PAN-PCN 0.6% nanofiber membrane (diameter: 14 mm) was added to the bottom of a beaker, then 3 mL absolute methanol solution containing 37 μM DPBF was slowly added. The beaker was illuminated by a 532 nm handheld laser (85 ± 1 mW/cm^2^) for 16 min, and the UV-visible absorbance spectrum of the DPBF methanol solution was recorded every 4 min.

### 2.7. Cell Viability Evaluation

The dark cytotoxicity of the as-fabricated PAN-PCN X% nanofiber membranes were evaluated by MTT assay. Dulbecco’s Modified Eagle’s Medium (Gibco, Waltham, MA, USA) supplemented with 10% fetal bovine serum (FBS), 100 μg/mL streptomycin and 100 μg/mL penicillin was used to culture L929 cells. In a typical procedure, the PAN-PCN X% nanofiber membranes were sterilized and L929 cells were seeded on the surface of the membranes. After 24 h of co-incubation, the medium was replaced by 0.1 mL MTT solution (0.5 mg/mL). The well plate was then incubated in a humidified incubator at a temperature of 37 °C, filled with 5% carbon dioxide. After 4 h incubation, 0.1 mL of the solution in each well was replaced by 0.1 mL of DMSO. After shaking slightly for about 10 min, 0.1 mL of the solution was then transferred into an another well plate. Finally, the absorbance of the solution in each well was collected by a microplate spectrophotometer at the wavelength of 570 nm (EPOCH2T). Cells seeded on a tissue culture plate (TCP) without any treatment were set as control. Each group was conducted in triplicate.

### 2.8. Live and Dead Cell Staining Assay

Live and dead cells were stained by diluted calcein-AM and propidium iodide (PI), respectively. In order to remove the excess serum, the cells were washed several times with PBS, then the calcein-AM solution was added in the well and incubated in the dark for 15 min. The calcein-AM solution was removed by washing for three times followed by adding PI solution. The well was further incubated in the dark for 5 min. Finally, the staining was ended by washing with PBS, and the results were observed on an FV1200 OLYMPUS fluorescence microscopy.

## 3. Results and Discussion

In this research, we fabricated a PCN-224 NPs embedded PAN nanofiber membrane (PAN-PCN) with high aPDI efficiency via electrospinning method. Due to the weight percentage difference of PCN-224 NPs in PAN, we prepared PAN-PCN 0%, PAN-PCN 0.1%, PAN-PCN 0.3% and PAN-PCN 0.6%. Firstly, characterization and discussion of the as fabricated nanofiber membrane are presented. 

**Figure 1:**
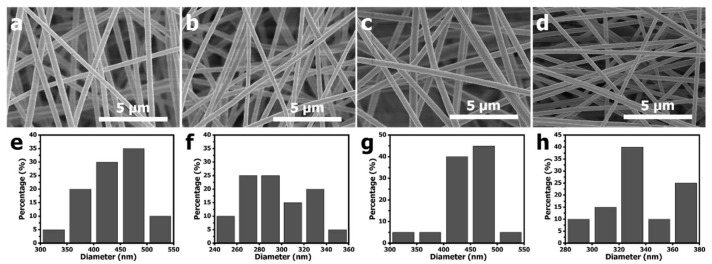
Scanning electron microscopy (SEM) images and diameter distributions of (**a**,**e**) polyacrylonitrile (PAN) 0%, (**b**,**f**) PAN-porous coordination network (PAN-PCN) 0.1%, (**c**,**g**) PAN-PCN 0.3% and (**d**,**h**) PAN-PCN 0.6% nanofiber membranes.

Figure 1 shows the SEM images and the diameter size distributions of electrospun PAN-PCN nanofiber with different amounts of PCN-224 NP loading. Compared with PAN-PCN 0% (Figure 1a), the surfaces of PAN-PCN 0.1%, PAN-PCN 0.3% and PAN-PCN 0.6% do not change (Figure 1b–d). We further randomly selected 20 nanofibers from each sample and calculated their diameter average value. The average diameters of PAN-PCN 0%, PAN-PCN 0.1%, PAN-PCN 0.3% and PAN-PCN 0.6% were 435, 293, 447 and 336 nm, respectively, and it seems no regular changes could be concluded. This reveals that the surface morphology and diameter size of the PAN-PCN nanofibers are not interrupted by incorporating PCN-224 nanoparticles via electrospinning.

**Figure 2:**
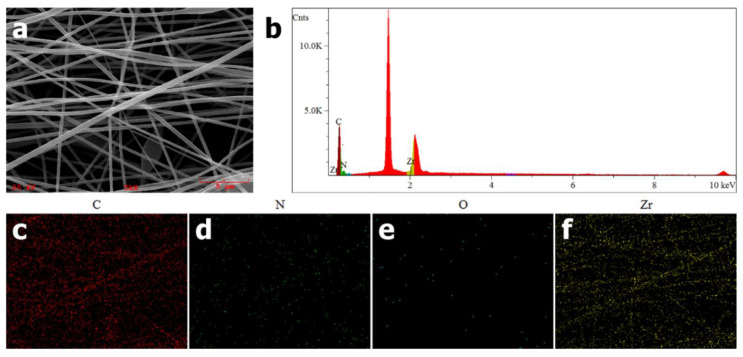
(**a**) SEM image and (**b**) energy-dispersive spectroscopy (EDS) results of PAN-PCN 0.6% nanofibers. (**c–f**) EDS mappings identifying the elemental distributions of C, N, O and Zr.

EDS was used to confirm the successful incorporation of PCN-224 NPs into PAN nanofibers. As shown in Figure 2c–f, as expected, uniform elemental distributions of carbon, nitrogen, oxygen and zirconium along the nanofibers could be observed. Furthermore, a signal peak at around 2.1 keV in Figure 2b is attributed to Zr element, which is a characteristic element of PCN-224 distinguishing from PAN. From the above EDS elemental analysis, PCN-224 NPs are proven to be introduced into PAN nanofibers preliminarily.

**Figure 3:**
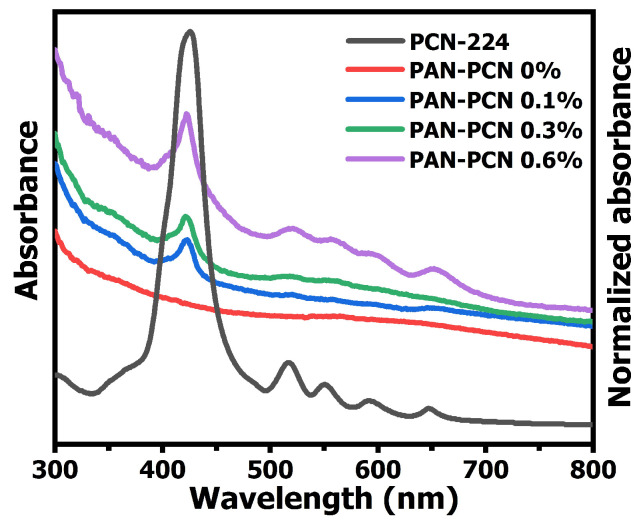
Absorbance spectra of PAN-PCN 0%, PAN-PCN 0.1%, PAN-PCN 0.3% and PAN-PCN 0.6%, as well as normalized absorbance spectrum of PCN-224.

UV-vis spectroscopy was also used to explore the absorbance spectra of PCN-224, PAN-PCN 0%, PAN-PCN 0.1%, PAN-PCN 0.3% and PAN-PCN 0.6%. The absorbance spectrum of PCN-224 was first recorded and analyzed. As shown in Figure 3, a strong intense peak at around 425 nm is attributed to the Soret band and another four relative weak peaks at around 516, 551, 591 and 647 nm belong to the Q bands [14]. We ascribe these absorption bands to porphyrin, which in this research are from TCPP. For the pure PAN nanofiber membrane, no obvious absorbance peak could be observed while after introducing PCN-224, a main strong peak at 422 nm and four new weak peaks at round 521, 559, 594 and 652 nm appear in the spectrum of PAN-PCN 0.6%, indicating the successful loading of PCN-224 nanoparticles into PAN nanofiber. However, for PAN-PCN 0.1% and PAN-PCN 0.3%, even the peak at 422 nm is obvious, and the peaks belong to the Q bands seems inconspicuous, which may be due to the low content of PCN-224 nanoparticles. 

**Figure 4:**
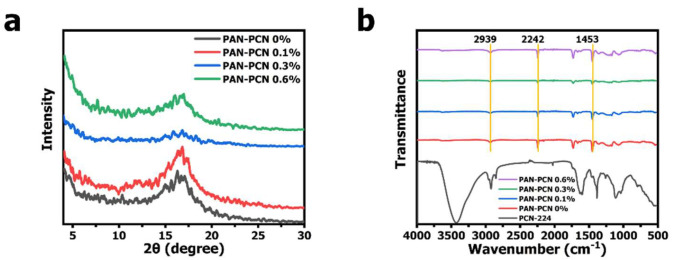
(**a**) X-ray diffraction (XRD) patterns of PAN-PCN 0%, PAN-PCN 0.1%, PAN-PCN 0.3% and PAN-PCN 0.6%. (**b**) FT-IR spectra of PCN-224, PAN-PCN 0%, PAN-PCN 0.1%, PAN-PCN 0.3% and PAN-PCN 0.6%.

In our previous work, diameter distribution and XRD analysis of PCN-224 NPs was presented [15]. It can be noted that PCN-224 NPs are well dispersed and the shape is spherical. Compared with PAN nanofiber size, the diameter of PCN-224 NPs is much smaller, distributing mostly from about 60 to 120 nm (average size: ~ 88 nm). The XRD pattern of PCN-224 reveals characteristic peaks at around 2θ = 4.62°, 6.54°, 7.90°, 9.12°, and 11.14°, which represents the crystal planes of (0 0 2), (0 2 2), (2 2 2), (0 0 4), and (2 2 4), respectively. These previous characterization results reveal that PCN-224 nanoparticles could be successfully synthesized. However, we further analyze the structure changes of PAN nanofibers after PCN-224 loading. From Figure 4a, PAN-PCN 0%, PAN-PCN 0.1%, PAN-PCN 0.3% and PAN-PCN 0.6% all have a diffraction peak at 2θ = 16.83°, which represents the (2 0 0) plane of PAN [16]. Interestingly, with the increase of PCN-224 NPs loading, the diffraction peak seems broad and weak. The reason may be the PCN-224 NP incorporation affected the orientation of the crystalline structure of the PAN nanofibers [16,17]. FT-IR transmittance spectra were collected to understand the groups of as-fabricated nanofiber membrane. As shown in Figure 4b of all the PAN-based nanofibers, the peak at 2939 cm^−1^ could be attributed to the stretching vibration of methylene (-CH_2_-), the nitrile position (-CN-) of pristine PAN nanofibers could be confirmed by the characteristic peak at 2242 cm^−1^, and the peaks at 1453 cm^−1^ confirm the bending vibration of methylene (-CH_2_-) [16]. The above XRD and FT-IR analyses show that the incorporation of PCN-224 NPs had little effect on PAN nanofibers. These results seem different from our previous work in which PCN-224 signals could be clearly detected from the cotton fiber surface [15]. The reason can be summarized as the different decoration methods of PCN-224 NPs. In this research, the NPs were embedded into the fiber during the electrospinning, which seems difficult to detect. The in situ growth method, however, allows the NPs to be anchored on the surface of the fibers, making them easier to detect.

**Figure 5:**
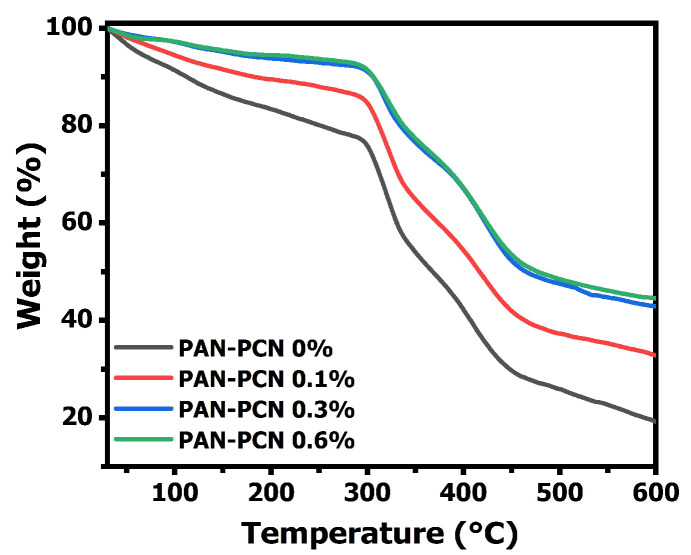
Thermogravimetric analysis (TGA) curves of PAN-PCN 0%, PAN-PCN 0.1%, PAN-PCN 0.3% and PAN-PCN 0.6%.

The TGA curves of electrospun PAN-PCN nanofiber membranes are shown in Figure 5, and all the composite fibers present good stability below 297 °C. However, the fibers suffer three sharp mass losses from 30 to 600 °C, which is related to the formation of rings (cyclization), decomposition and carbonization of PAN, respectively [18]. Interestingly, the results indicate that the weight loss curves drop slowly with the increasing loading of PCN-224 NPs. This means that the surface adhesion between the NPs and the PAN polymer is beneficial to the thermal stability enhancement. The reason might be due to the strong interaction between the electronegative groups from the PAN chains and the PCN-224 NPs [19,20]. 

**Figure 6:**
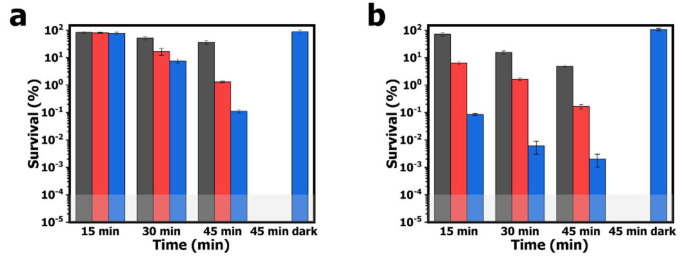
Antibacterial photodynamic inactivation (aPDI) studies employing PAN-PCN 0.1% (dark grey column), PAN-PCN 0.3% (red column) and PAN-PCN 0.6% (blue column) against (**a**) *E. coli* and (**b**) *S. aureus* for different illumination times.

The aPDI study employing the electrospun PAN-PCN nanofibers was performed with model bacterial strains of *E. coli* and *S. aureus*, and the survival rates of bacterial cells are shown in Figure 6. After 60 min of pre-incubation and 15 min of illumination, the survival rates of *E. coli* cells treated by PAN-PCN 0.1%, PAN-PCN 0.3% and PAN-PCN 0.6% are 83.5% (*p* = 0.93, ~ 0.08 log unit), 80.4% (*p* = 0.71, ~ 0.09 log unit) and 76.9% (*p* = 0.45, ~ 0.11 log unit), respectively. The results reveal that only a small number of *E. coli* cells were inactivated. However, the survival rates of *S. aureus* cells are 71.3% (*p* < 0.05, ~ 0.15 log unit), 6.4% (*p* < 0.001, ~ 1.19 log unit) and 0.1% (*p* < 0.001, 3.00 log unit), respectively (Figure 6b). It is noteworthy that different inactivation efficiencies between *E. coli* and *S. aureus* cells exist, and this is attributed to the special lipopolysaccharide (LPS), which is a highly impermeable extra layer of the Gram-negative bacteria strains [21]. LPS makes Gram-negative bacterial cells more aPDI-resistant than Gram-positive bacterial cells. After 30 and 45 min of illumination, the bacterial inactivation rates of the material are increased. Under 45 min of illumination, the survival rates of bacterial cells treated with PAN-PCN 0.6% are the lowest, and the survival rates of *E. coli* cells are 0.1% (*p* < 0.01, 3.00 log unit); however, *S. aureus* cells were inactivated by 99.9+% reduction (*p* < 0.001, ~ 4.70 log unit). Although the as-fabricated PAN-PCN nanofiber membrane exhibits desirable aPDI efficiency under illumination, we still need to rule out the possibility of phototoxicity from PAN and dark cytotoxicity from PAN-PCN nanofibers towards bacteria. However, according to our previous study [22], absolute PAN nanofibers exhibited no obvious phototoxicity to bacteria. In the absence of light, the survival rates of *E. coli* and *S. aureus* cells treated by PAN-PCN 0.6% for 45 min (45 min dark in Figure 6) are 87.8% and 106.5%, respectively, which further confirms a lack of dark cytotoxicity. 

**Figure 7:**
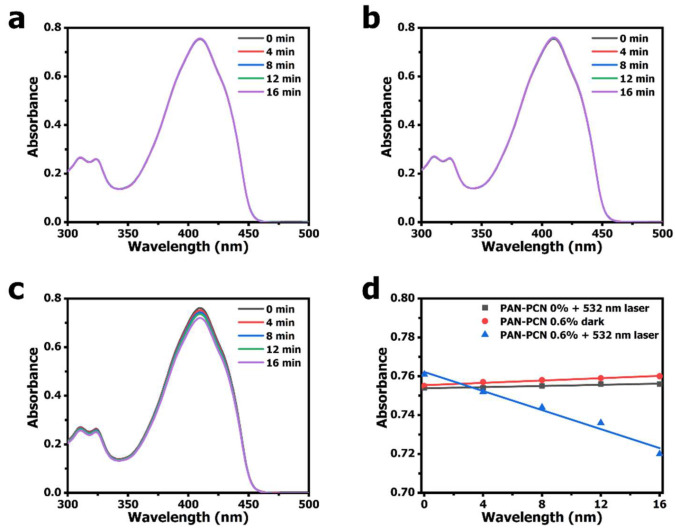
Absorbance spectra of DPBF (1,3-diphenylisobenzofuran) in the presence of PAN-PCN 0.6% under illumination. The spectrum curves were collected every 4 min under a handheld laser. (**a**) DPBF absorbance spectra in the presence of PAN-PCN 0% under laser illumination. (**b**) DPBF absorbance spectra in the presence of PAN-PCN 0.6% without illumination. (**c**) DPBF absorbance spectra in the presence of PAN-PCN 0.6% under laser illumination. (**d**) DPBF photooxidation rate constant, the absorbance values at wavelength of 410 nm were collected from panel (**a**–**c**).

According to other research, porphyrinic MOFs could be used as effective PSs for ^1^O_2_ generation [8,23,24]. The mechanism is as follows: photosensitive materials absorb light energy forming an excited singlet state, then give off fluorescence. Moreover, intersystem crossing happens and PS molecules form an excited triplet state. Finally, ^1^O_2_ is generated through the energy transfer process between the unstable excited triplet state photosensitive materials and the ^3^O_2_ in ground state [25]. We measured the ability of the ^1^O_2_ generation of PAN-PCN 0.6% under 532 nm laser illumination. DPBF, a sensitive ^1^O_2_ trapping agent, was used to detect ^1^O_2_ generation based on the main absorbance peak at 410 nm [26,27]. From Figure 7c, the obvious decrease of DPBF absorbance at 410 nm with the increasing illumination time reveals the ^1^O_2_ generation of PAN-PCN 0.6%. However, when performed in the presence of PAN-PCN 0% with laser or in the presence of PAN-PCN 0.6% without laser, no notable reduction of DPBF absorbance is recorded. In brief, the ^1^O_2_ detection study proves the ^1^O_2_ formation from PAN-PCN nanofiber membrane under visible light illumination.

**Figure 8:**
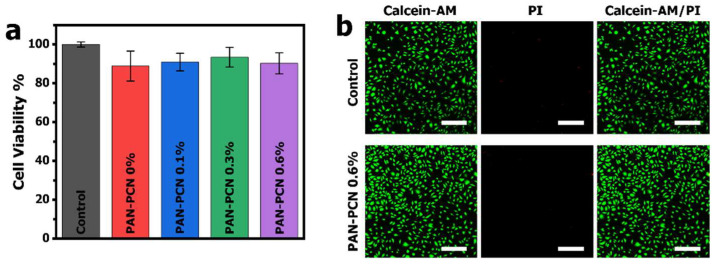
(**a**) Viability (%) of L929 cells treated by PAN-PCN 0%, PAN-PCN 0.1%, PAN-PCN 0.3% and PAN-PCN 0.6% after co-incubation for 24 h. (**b**) Fluorescence microscopy images of L929 cells treated by PAN-PCN 0.6% after 24 h co-incubation. The scale bars inserted in panel (**b**) are 300 μm.

MTT assay was applied to evaluate the cytotoxicity of PAN-PCN 0%, PAN-PCN 0.1%, PAN-PCN 0.3% and PAN-PCN 0.6%, L929 cells were seeded on the surface of the nanofiber membranes. After 24 h of co-incubation, the cell viability results are shown in Figure 8. From Figure 8a, the survival rates are all above 85% and there is no regular variation between cell survival rate and the loading of PCN-224 NPs. In order to observe the morphology of the cells after being treated by the PAN-PCN nanofibers, fluorescence microscopy was used to image the L929 cells. As shown in Figure 8b, the L929 cells without treatment with the materials reveal mostly green fluorescence, which indicates the survival of healthy cells. For PAN-PCN 0.6% treated cells, as expected, only a few red fluorescence points could be observed, indicating desirable biocompatibility. These results also demonstrate the aPDI result of a lack of dark cytotoxicity towards bacterial cells. Taken together, the as-fabricated PAN-PCN nanofibers present good biocompatibility.

## 4. Conclusions

In summary, photosensitive MOF material, PCN-224 NPs, were synthesized by solvothermal method and then embedded into PAN nanofibers successfully by electrospinning. PAN nanofibers could not only help to avoid drug leakage, but also be used as a flexible support material to load PCN-224 NPs. The aPDI study revealed that the PAN-PCN 0.6% nanofiber membrane exhibited effective elimination of *E. coli* and *S. aureus* at low PCN-224 loading concentration. The mechanism study demonstrated that the good aPDI efficiency was due to the strong oxidation of ^1^O_2_. Even though the materials possessed strong bactericidal activity, the MTT assay demonstrated that the as-fabricated nanofiber membranes had good biocompatibility under dark conditions. Taken together, this research has provided a means of using phototoxic composite nanofiber membranes against bacteria, which could be used to meet the challenge of drug resistance and spread of pathogens.

## Data Availability

Not applicable.
